# Considerations for shared decision-making in treatment of chronic rhinosinusitis with nasal polyps

**DOI:** 10.3389/falgy.2023.1137907

**Published:** 2023-03-10

**Authors:** Shreya P. Ramkumar, Devyani Lal, Amar Miglani

**Affiliations:** ^1^ Department of Otolaryngology – Head and Neck Surgery, Mayo Clinic Hospital, Phoenix, AZ, United States; ^2^ Saint Louis University School of Medicine, Saint Louis, MO, United States

**Keywords:** chronic rhinosinusitis (CRS), shared decision making, biologics, nasal polyps (NP), treatment

## Abstract

Shared decision-making is an approach where physicians and patients work together to determine a personalized treatment course. Such an approach is integral to patient-centered care in chronic rhinosinusitis with nasal polyps (CRSwNP). CRSwNP is a chronic inflammatory condition of the sinonasal cavity that can severely impact physical health, smell, and quality of life (QOL). Traditional standard-of-care treatment options include topical (i.e. sprays) and oral corticosteroids and endoscopic sinus surgery, but more recently, novel corticosteroid delivery methods (i.e. high volume irrigations, recently-approved exhalation breath-powered delivering devices, and drug-eluting steroid implants) and 3 new FDA approved biologics directed against type II immunomodulators have become available. The availability of these therapeutics offers exciting new opportunities in CRSwNP management but requires personalized and shared-decision making as each modality has variable impacts on CRSwNP and related comorbid conditions. Studies have published treatment algorithms, but the practical use of these lean guidelines is heavily influenced by the lens of the treating physician, the most common being otolaryngologists and allergy immunologists. Clinical equipoise occurs when there is no basis for one intervention to be regarded as “better” than another. While most guidelines, in general, support the use of topical corticosteroids with or without oral corticosteroids followed by ESS for the majority of unoperated CRSwNP patients, there are situations of clinical equipoise that arise particularly in CRSwNP who have failed surgery or those with severe comorbid conditions. In the shared decision-making process, clinicians and patients must consider symptomatology, goals, comfort, compliance, treatment efficacy, and treatment cost when determining the initial choice of therapy and escalation of therapy with the potential use of multiple modalities for recalcitrant CRSwNP. A summary of salient considerations that might constitute shared decision-making is presented in this summary.

## 1. Introduction

Shared decision-making (SDM) is an approach by which physicians and patients work together to develop a personalized treatment plan by combining evidence-based medicine with patient values. This approach has been shown to improve patient satisfaction, reduce decisional conflict, and increase adherence to treatment (1–4). One area of SDM application is in treatment options for chronic rhinosinusitis with nasal polyps (CRSwNP), a subset of chronic rhinosinusitis (CRS).

The growing number of effective treatments for CRSwNP is a cause for optimism but raises questions in both patients and clinicians on which treatment to pursue. Currently approved therapeutics by the Food and Drug Administration (FDA) for indications of nasal polyps include corticosteroid nasal sprays (including the novel exhalation breath-powered corticosteroid devices), three monoclonal antibodies for biological therapy, and corticosteroid-eluting absorbable sinus implants. In addition, oral corticosteroids, as well as topical corticosteroid nasal rinses, are commonly utilized for the treatment of CRSwNP, as is endoscopic sinus surgery (ESS). Studies have presented a stepwise algorithm of guidelines on options of treatment for CRSwNP (5), but no universally accepted guidelines are available. In CRSwNP, treatment has been classically divided into upfront management followed by maintenance treatment. Oral corticosteroids, ESS, and biologics have been proposed as options for the up-front treatment of nasal polyps whereas topical intranasal corticosteroids and biologics are options for maintenance treatment (6, 7). However, endpoints for cessation of maintenance treatment, particularly biological therapy, have not been defined. Approved biological therapies for nasal polyps include omalizumab, [a monoclonal antibody to immunoglobulin E (IgE)], mepolizumab [a monoclonal antibody to interleukin-5 (IL-5)], and dupilumab [a monoclonal antibody to the IL-4 receptor alpha subunit, blocking IL-4 and IL-13 activity]. Currently, there are neither head-to-head trials nor guidelines available to help decide between these FDA-approved biological treatments.

The debate on the advantages and disadvantages of treatment options is complex and dependent on factors such as which symptoms are bothersome, goals of treatment, efficacy, treatment size effect, comfort with specific risks associated with each modality, compliance issues, and treatment cost. On the horizon is the development of prognostic and therapeutic biomarkers which will also impact the choice of treatment, allowing for a shift towards a more personalized approach. SDM's integration into Otorhinolaryngology has not been widely adopted but a paradigm shift in the management of CRSwNP utilizing SDM is imperative and imminent (8).

The purpose of this paper is to summarize important SDM considerations in CRSwNP treatment. We will present applications of SDM in deciding treatment and assessing risks, outcomes, cost, and delivery of care. Barriers to SDM and the role of decisional aids will then be discussed.

## 2. Clinical applications of SDM

### 2.1. What is SDM and when should it be used?

Although the definition of SDM is standardized, the clinical implementation of SDM is much more complex. Three essential elements must be present. First, two or more participating parties should acknowledge that a decision must be made (9). Second, all parties should understand the risks and benefits of each option (9). Third, a mutual decision is agreed upon by all parties (9). Based on these three key principles, multiple conceptual models of SDM have been developed. A systematic review found that there were at least 40 models with 24 overarching clustered components (10). Although more data on the framework most suitable for use in CRSwNP is needed, these models provide a structure for implementation.

Clinical equipoise occurs when there is no basis for one intervention to be regarded as “better” than another (11). Clinical equipoise of treatment options for CRSwNP can lead to uncertainty of treatment course and decisional conflict (2). In such cases, SDM is a powerful tool that can be utilized. Decisional conflict is defined as “uncertainty about which course of action to take when choice among competing options involves risk, loss, regret, or challenge to personal life values” and is a key element in decision making (12). Both clinical equipoise and decisional conflict are prevalent concepts in the clinical decisions within rhinology as many of the procedures offered are elective and many treatments offer similar outcomes (5, 13).

Table 1 summarizes the multiple treatment options available for CRSwNP including indications, advantages, and disadvantages of each. Table 2 highlights some of the important clinical factors and considerations to keep in mind when incorporating SDM for the treatment of CRSwNP. Finally, Table 3 highlights some hypothetical scenarios for which SDM can be implemented.

**Table 1:** Summary of the indications, advantages, disadvantages, cost, and other considerations for various treatment options for patients diagnosed with chronic rhinosinusitis with nasal polyps.

Treatment:	Corticosteroid nasal spray	Corticosteroid saline rinse	Exhalation delivery system with fluticasone	Sinus implant	Biologics (Dupilumab, Omalizumab, Mepolizumab)
How are they used	Nasal spray	Compounded corticosteroid mixed with saline. Examples include NeilMed rinse bottle Nettipot, but multiple delivery devices exist.	Breath powered delivery	Small steroid-coated implant (also referred to as a stent) is placed within your sinus and slowly releases medication over weeks	Self-injected medication under the skin every 2–4 weeks
When are they an option	First-line	First-line	First-line, approved to treat nasal polyps specifically	Recurrence, persistence of symptoms, or postoperative management after sinus surgery	Recurrence or persistence of symptoms after sinus surgery or contraindication to surgery
Advantages of treatment	Simple to use, inexpensive, over the counter	Simple to use, inexpensive, over the counter	Simple to use, reaches certain areas of sinuses better than sprays or rinse	Performed in doctor's office	Treatment option promising for patients who cannot undergo surgery
Disadvantages of treatment	Symptoms from treatment including burning, crusting, and bleeding in the nose	Symptoms from treatment including burning, crusting, and bleeding in the nose	Prescription needed for use, requires breathing coordination	May need repeated procedures	Expensive, may need to continue therapy long-term for relief
Treatment cost^a^	$	$	$$	$$–$$$	$$$$
Other important information	May be used concurrently with another therapy	May be used concurrently with another therapy	Must be used every day and only available in one type (fluticasone)	Previous sinus surgery must have been performed on the ethmoid sinus to be eligible.	Relatively new therefore no information on long-term outcomes

^a^ $ indicates a relative range of cost with $ in reference to lower cost and $$$$ to more expensive cost.

Adapted from Shared Decision-Making Tools developed by the American Rhinologic Society and American College of Allergy, Asthma, and Immunology (94,95).

**Table 2:** Critical factors of consideration for various treatment options available for chronic rhinosinusitis with nasal polyps.

	Critical factors in shared decision making (SDM) for chronic rhinosinusitis with nasal polyps				
		When to use	Clinical equipoise and uncertainty	Treatment outcomes and symptom importance	Comorbid conditions to consider
1	*Corticosteroid nasal spray/saline rinse*	First-line	Can use in combination with other treatment modalities, over the counter and cheap	Possible polyp size reduction, improvement in nasal congestion, loss of smell, postnasal drip, and rhinorrhea	Contraindications to use of corticosteroids
	*Exhalation delivery system with fluticasone*	First-line	Larger corticosteroid dose than nasal sprays, patient requires prescription for use and treatment is costlier	Enhanced reach of medication to areas of sinuses, rhinorrhea, facial pain/pressure, smell, and nasal polyp size reduction	Lung disease or other conditions that may affect breathing coordination, contraindication to corticosteroids
2	*ESS*	Escalation of treatment	Majority of patients, invasive procedure	Reduction of polyp size	Heart disease, lung disease, and other contraindications to surgery including anesthesia reactions
	*Biologics*	Escalation of treatment and disease refractory to ESS	Minority of patients, requires long-term continuation of medication	No data available on long-term outcomes, may be helpful in decreasing polyp size, and improving sinus opacification, nasal congestion, and anosmia	Financial burden to patient, side effects of biologics that may exacerbate medical conditions, unwillingness to self-inject
3	*Sinus implant*	Postoperative management or disease refractory to ESS, surgery must have been performed in the ethmoid sinus	Can use in combination with other treatment modalities	Reduction of nasal polyp grade and nasal congestion	Cannot undergo multiple in-office procedures

ESS, endoscopic sinus surgery.

**Table 3:** Example hypothetical scenarios and respective treatment options available for which shared decision-making may be utilized .

Hypothetical scenario	Treatment options for which SDM can be used to arrive at a plan
Patient has contraindication to corticosteroids	ESS, biologics
Patient does not have short-term cash available for medical expenses and does not have insurance	Corticosteroids (multiple formats of delivery)
Patient has contraindications to anesthesia	Corticosteroids, biologics
Patient has symptomatic recurrence of nasal polyps after revision ESS	Revision ESS, biologics, (with or without sinus implants/corticosteroids)
Patient has transportation issues and cannot make scheduled appointments regularly	Corticosteroids, ESS, biologics
Patient prefers not to take medication multiple times a day	Biologics, ESS, sinus implant
Patient is afraid of needles and does not want surgery	Corticosteroids (multiple formats of delivery)
Patient's primary goal is reduction or elimination of nasal polyps	ESS, corticosteroids, biologics
Patient's primary goal is improvement of smell	Biologics, corticosteroids, ESS

Of note: treatment options available may also be combined.

ESS, endoscopic sinus surgery; SDM, shared decision making.

### 2.2. Treatment indications and risk

#### 2.2.1. Topical corticosteroids

Corticosteroids are useful in suppressing chronic inflammation and have long been recommended as a treatment modality for CRSwNP both in oral and topical nasal forms. They are the mainstay of maintenance medical therapy for CRSwNP. Importantly, most biological therapies approved for nasal polyps used intranasal corticosteroid sprays in the control arm as a standard of care (14–17). In reviewing data from randomized clinical control trials, intranasal corticosteroids appeared to improve symptoms of nasal congestion, rhinorrhea, and sneezing and have a very modest effect on polyp size (6, 7). According to the European Position Paper on Rhinosinusitis and Nasal Polyps 2020 (EPOS 2020), self-care and primary care treatment for patients presenting with two or more CRS symptoms includes saline spray/rinse and intranasal corticosteroids (6). Appropriate medical therapy for diffuse and bilateral CRS also primarily refers to corticosteroid nasal sprays/drops/rinses along with saline rinses (6). Topical intranasal corticosteroids as sprays or as part of nasal rinses appear to be generally safe for use in adults (18). Importantly, intranasal corticosteroids can be used concurrently with other treatment modalities in refractory disease.

#### 2.2.2. Systemic corticosteroids

The International Consensus Statement on Allergy and Rhinology from the American Rhinologic Society (ARS) also includes oral corticosteroids (OCS) in the management of CRSwNP. OCS are inexpensive and although they can be effective in shrinking polyp size and improving symptoms of nasal obstruction and smell loss, there are limitations in the duration and usage of these OCS due to the risk of significant systemic side effects such as avascular necrosis of the femoral head, weight gain, cataracts, osteoporosis, increased risk of infection, mood changes, glaucoma, and acid reflux (19). Based on a survey of Italian rhinologists (*n* = 437) who regularly manage CRSwNP patients, the most frequently observed adverse events encountered with the administration of systemic steroids were hypertension (57.6%), hyperglycemia (55.76%), insomnia (50%), acid reflux (29.44%), anxiety (23.27%), diabetes (23.04%), mood changes (21.66%), increased appetite (12.67%), and glaucoma (10.83%) (19).

A prospective trial evaluating the hypothalamic-pituitary-adrenal axis of bilateral nasal polyposis patients (*n* = 46) who had a minimum of three courses of systemic steroids (1 mg/kg/day) in the past year found 10.9% of their cohort had osteoporosis, 43.5% had osteopenia, and 48.8% of tested patients had asymptomatic adrenal insufficiency (*n* = 20) (20). A retrospective study of CRSwNP patients (*n* = 197) receiving 5 mg of steroids for 3 consecutive months found low bone mineral density in 38.6% of the patients (21). An association between cumulative steroid doses with loss of bone density was identified (21). Additionally, steroid users have been found to have a 1.8 times higher risk of presenting with upper gastrointestinal complications than nonusers (22). Several cardiovascular risks have also been shown with the use of steroids, particularly in dosages higher than 7.5 mg (23, 24). The European Academy of Allergy and Clinical Immunology reviewed the available literature on the benefits and harms of systemic steroid treatment for rhinitis and rhinosinusitis and recommended that steroids should not be considered as first-line treatment but may be used in a short course of 2–3 weeks (23). OCS, therefore, is recommended for severe symptomatology and for acute exacerbation of CRSwNP. EPOS 2020 suggests that 1–2 courses per year may be useful for patients with partial or uncontrolled disease (6). However, maintenance therapy with OCS is not recommended.

#### 2.2.3. Exhalation delivery system of fluticasone

The exhalation delivery system of fluticasone (EDS-FLU) is an FDA-approved treatment option for nasal polyps. EDS-FLU may be used prior to moving the patient along to surgery or biological therapy (5). This corticosteroid delivery method may be used for both primary nasal polyps as well as in maintenance treatment, although it has also been used for CRS patients without nasal polyps. According to Han et al., EDS-FLU is recommended for the persistence of symptoms after intranasal steroid sprays (5). A meta-analysis of 61 randomized control trials critically evaluating the delivery of corticosteroids found that EDS-FLU improved health-related sinonasal outcome test (SNOT-22) scores by a mean of 7.86 points, reduced nasal obstruction by 0.35 (scale 0–3) improved smell by 4.10 (scale 0–40), and reduced nasal polyp size by 0.56 (scale 0–3) (25). Translated, patients will likely experience a modest improvement in nasal obstruction symptoms and polyp size (25). There is low certainty of evidence that suggests that the quality-of-life outcomes including SNOT-22 and smell may improve slightly (25).

Results from the EXHANCE-12 trial measuring efficacy and safety in a 12-month timeline found that adverse event rates tended to decrease or resolve spontaneously with continued EDS-FLU use (26). In terms of efficacy among nasal polyp patients, 54.2% had polyp elimination in at least one nostril while 83.3% had improvement of polyp grade by one or more points (26). After 12 months of treatment, the baseline bilateral polyp score decreased from 2.8 to 1.3. Mean SNOT-22 scores also decreased with a magnitude of improvement similar among CRSwNP (−21.5 at 12 months) and CRS without nasal polyps (−21.1 at 12 months) patients (26). For optimal benefits of EDS-FLU use, breathing coordination is a necessary pre-requisite. The safety profile of EDS-FLU (372 µg BID) is like that of conventional intranasal corticosteroid sprays and is well-tolerated (26, 27). Of 705 patients with CRS enrolled in the EXHANCE-3 prospective 12-week cohort study, adverse events occurred for 22 participants (27). The most common adverse events reported were nasal mucosal disorder (10.2%), epistaxis (6.8%), nervous system disorder (5.7%), and nasal septum disorder (5.5%) (27).

#### 2.2.4. Corticosteroid-eluting sinus implants

Bioabsorbable steroid-eluting sinus implants are an in-office treatment option available for CRS patients who are at least 18 years of age and have had prior ESS. Office-based drug-eluting implants have been approved for use in patients that have had prior ethmoidectomy or frontal sinusotomy and have recurrent polyps (28). A major advantage of sinus implants is the localized delivery of corticosteroids to areas of inflamed tissue (29). A recent survey following the Delphi process highlighted critical statements that expert clinicians reached a consensus on regarding indications for use of sinus implants (30). Majority of the otolaryngologists surveyed (*n* = 14) agreed that steroid-eluting stent placement should be considered regardless of polyp status when patients had extended frontal sinus surgery, are intolerant of oral steroids, are diabetic, or have recurrent stenosis (30). No consensus was reached regarding the consideration of sinus implants in poorly compliant patients after ESS or as an alternative to biologics for recurrent CRSwNP (30). The drug-eluting absorbable devices may be used prior to moving the patient along to biological therapy or during the postoperative management period (5). The lack of expert consensus on the use of sinus implants demonstrates the increased utility of SDM in developing patient-centered treatment options (30).

In a cohort of 20 prospectively followed patients who had placement of a sinus implant in an office setting, only 2 adverse events occurred. Both events were cases of acute sinusitis that resolved with antibiotics (31). Additionally, the office placement procedure was found to be well tolerated by patients (31). Importantly, it was revealed that the mean ethmoid sinus inflammation was reduced from 25.6 to 18.9 by four weeks, a statistically significant change, and SNOT-22 scores also significantly improved. The treatment effect size was 1.38 at two weeks and 1.91 at four weeks representing a clinically significant improvement (31). A recent meta-analysis determined that delivery of corticosteroids by stent was most beneficial in the reduction of nasal obstruction symptoms (mean change −0.31 on a scale of 0–3), and subjective smell function (mean change of 3.81 in a scale of 0–40) (25). Previous studies that analyzed systemic safety found that indwelling steroid-eluting stents would likely not have adrenal-pituitary axis suppression effects and that sinus implants were a safe and effective therapeutic option (32).

#### 2.2.5. Biologics

Most recently, the use of biologics has dominated the conversation of SDM and clinical equipoise for CRSwNP. Studies have postulated that biologics are favorable options in recalcitrant disease such as in those who may fail aspirin desensitization or who require repeat systemic corticosteroids (13, 33). Additionally, patients suffering from comorbidities that biologics can treat, such as atopic dermatitis and chronic urticaria, might likewise benefit from this treatment choice (34, 35). In patients with comorbid asthma, dupilumab with mometasone furoate nasal spray improved lung function as measured by forced expiratory volume and subjective control in asthma tests which exceeded the minimally clinically important difference (36). In patients with comorbid atopic dermatitis and CRS, dupilumab improved the eczema area and severity index score from 34.2 to 4.3 at 16 weeks (36). Additionally, the dermatology life quality index scores also significantly decreased from 24.0 to 4.0 (36). Similar promising results were seen for patients with chronic spontaneous urticaria, although only three patients were in the study cohort (36). Additional studies with larger patient cohorts characterizing the role of comorbidities in biologics for CRS are necessary.

Recently, Scadding et al. suggested a few considerations for using biologics in CRSwNP (37). Major considerations to begin biologics include type 2 inflammatory polyps, SNOT-22 score >40 points despite good use of corticosteroids and previous surgery, and use of corticosteroids in the past 12 months (37). Major consideration to continue biologics was proposed as improved quality of life or a greater than 50% reduction in systemic corticosteroid use without further surgery (37). EPOS 2020 also suggested similar indications for biologics use in CRSwNP and recommended that at least three of five pre-specified criteria should be met for use (6). However, CRSwNP is not a homogenous disease in which patient selection can be determined based on established cut-offs. In addition to the pre-specified criteria from EPOS 2020 which includes evidence of type 2 inflammation, need for systemic corticosteroids or contraindication, impaired quality of life, loss of smell, and asthma comorbidity (6), there are many other factors that should be considered such as the high financial cost of biologics, previous sinus surgery data, and the discrepancy of polyp grade within each nostril. Importantly, nasal endoscopy should show bilateral polyps for use of biologics (38). With so many variables of consideration, SDM is a powerful tool that can be utilized to develop a patient-centered treatment plan.

Additionally, it is important to note that the risks and benefits of each biological therapy available vary. Dupilumab targets the IL-4 alpha chain preventing cytokines of the Th2 response (36). Omalizumab is a monoclonal antibody against IgE. Omalizumab binds to free IgE reducing the availability and allowing neutralization of the inflammatory pathway without degranulation (39). Finally, mepolizumab is a humanized monoclonal antibody specific for IL-5 and reduces blood eosinophil counts (17). Based on these various mechanisms of action, the appropriate biologic can be found utilizing patient factors. For example, a patient with an eosinophilic CRS endotype may preferentially benefit from mepolizumab. In this avenue, it is also important to consider specific biomarkers that may be helpful in utilization for shared decision-making. Examples of such biomarkers include serum eosinophil counts, tissue eosinophil counts, serum IgE levels, and antineutrophil cytoplasmic antibodies. Interestingly, dupilumab treatment was found to decrease biomarker concentrations in nasal secretions and nasal polyp tissue including those of eotaxin-2, eotaxin-3, IgE, and IL-13 (40). Biomarkers signify the importance of patient selection in considering treatment recommendations and are at the forefront of the shift toward personalized medicine in CRSwNP. Currently, however, the specificity and sensitivity of these markers in CRS and their role in treatment are still being developed to offer evidence-based conclusions.

A systematic review and meta-analysis (*n* = 13 studies) analyzed the adverse events of dupilumab, omalizumab, mepolizumab, and reslizumab in CRSwNP patients (41). Dupilumab trials reported pharyngitis, erythema, and headache as the most common adverse reactions. Omalizumab trials reported headaches, pharyngitis, and injection-site reactions. Mepolizumab and reslizumab reported complications of nasal polyps, congestion, pharyngitis, and infections as their highest adverse effects (41). The reported risk variations between each biologic may be important consideration to patients, especially those facing multiple medical comorbidities.

#### 2.2.6. ESS

For most patients with CRSwNP that show persistent polyps and symptoms despite appropriate medical management, ESS is offered as a standard of care. The extent of sinus surgery historically has been determined by the surgeon's discretion. However, increasingly mucosa-preserving wide-hole surgeries with “full house ESS” treating all paranasal sinuses are being adopted to facilitate postoperative medical management with intranasal corticosteroids delivered through high-volume saline nasal irrigations. It is possible that SDM can be utilized in sharing surgical extent for patient-centered care by incorporating patient values in the development of a treatment plan. Additionally, it is also important to share with patients the need for widely marsupialized ethmoidal corridors with a thorough dissection of ethmoidal septations of the lamina papyracea and skull base. ESS does not “cure” CRSwNP but is a treatment modality that may be particularly effective for removing large disease burden with polyps and mucinous debris which facilitates disease control by optimizing topical drug delivery to the sinonasal mucosa *via* wide ethmoidal corridors and large surgically created ostia. Patients with CRSwNP may need additional surgeries in the future, particularly if ethmoidal partitions are not thoroughly dissected and natural sinus ostia are not appropriately incorporated into surgically created wider openings.

Risks of ESS include the need for general anesthesia, postoperative infection, blood loss, and iatrogenic injury to the orbit, skull base, orbital contents, and intracranial cavity. A retrospective clinical study analyzing complications of ESS for CRS from one academic surgeon's 25-year practice (*n* = 3,402 patients) found the most common complications to be postoperative hemorrhage (37 cases), orbital complications (29 cases), and cerebrospinal fluid (CSF) leak (19 cases) (42). Additional important complications to be aware of include blindness, brain injury, diplopia, cheek hematoma, and meningitis (42). In a retrospective review of a nationwide database (*n *= 63,823 patients) who underwent ESS, complications included CSF leak (0.17%), orbital injury (0.07%), and hemorrhage (0.76%) (43). Intracranial complications (CSF leak), orbital injury (blindness), and anosmia were found to be the most common complications that resulted in lawsuits after ESS (44). The important anatomic structures that surround the paranasal sinuses will always confer a certain amount of risk during surgery and contributes to the complications seen both intraoperatively and postoperatively. Contraindications for ESS are poorly controlled medical conditions that preclude safe general anesthesia, patient aversion to surgical intervention, and unfavorable anatomy. For such patients with recalcitrant disease, corticosteroid eluting stents, EDS-FLU, or biologics may be an option.

### 2.3. Treatment delivery and outcomes

#### 2.3.1. Topical corticosteroids

There are many types of corticosteroid delivery options for CRSwNP including nasal sprays, oral, nebulization, steroid-eluting stent delivery, and direct infiltration.

Corticosteroid nasal sprays and rinses can be found over the counter at drugstores and offer the flexibility for patients to refill as needed without additional appointments with their provider. Patients who may not be able to visit their provider's clinic due to lack of transportation or work-hour restrictions may still be able to benefit from this treatment option. Intranasal corticosteroids can be classified into first and second generation which differ in bioavailability (45). A Cochrane Review of nine randomized control trials with a minimum three-month follow-up suggested that all intranasal corticosteroid sprays were similarly effective (46). The low-quality evidence available, heterogeneity in outcomes analyzed, and unclear significance in the small size of improvement, advocate the need for further analysis to determine if clinical significance exists. A meta-analysis of 61 randomized controlled trials (*n* = 7,176 patients) found that intranasal corticosteroid rinses improved sinusitis-related quality of life and nasal obstruction symptoms when compared to a placebo. Nasal obstruction had a mean −0.51 difference (scale 0–3) from baseline and was determined to be a beneficial treatment option (25). Additionally, subjective smell improved by a mean of 3.24 (scale 0–40), and polyp size reduced by a mean difference of 0.64 (scale 0–3) (25). Previous studies have found that steroids can help reduce polyp size but may not eradicate polyps (47). Delivery of corticosteroids through nasal spray and rinses may not be sufficient for medication to reach areas such as the frontal sinus. When comparing corticosteroid nasal irrigations to sprays, a randomized control trial (*n* = 44 patients) found that delivery by nasal irrigation was superior to nasal sprays in post-surgical CRS patients (48). Overall, one study suggests that 3.95 mm ostial diameter or greater maximizes the potential for appropriate delivery of corticosteroids to the paranasal sinuses in treatment (49). In cadaveric models, a 4.7 mm ostium size was determined to offer maximal penetration and delivery for the maxillary and sphenoid sinuses (50).

Atomizers and nebulizers are alternative delivery systems used for improved medication penetration. These devices atomize medication into particles of 30 to 100 µm that are then distributed intranasally (45). OCS provides a systemic effect rather than a localized one which may be beneficial to patients with other medical comorbidities. In a study analyzing short-term glucocorticoid application on CRSwNP patients (*n* = 127 patients), there were significant differences in outcomes based on the delivery of corticosteroids in a nasal spray, nasal drop, and orally (51). After treatment, the nasal polyp endoscopic score was reduced in the budesonide nasal drop (−0.82) and oral steroid group (−0.85) in comparison to the regular nasal spray group (−0.10) (51). Interestingly, the reduction in nasal polyp score was more significant in patients with eosinophilic CRSwNP and patients with greater than 30% tissue eosinophil infiltration representing the importance of patient selection factors that may impact treatment outcomes (51).

#### 2.3.2. Systemic corticosteroids

In a study of 17 patients who underwent combined steroid therapy (local and systemic), there was a statistically significant decrease of symptom scores and polyp size (47). Nasal polyp stage mean of 2.12 was lowered to a mean of 1.0 after treatment (47). Additionally, treatment with combined steroid therapy also improved nasal obstruction (pretreatment mean: 2.65, posttreatment mean: 0.88), rhinorrhea (pretreatment mean: 1.94, posttreatment mean: 0.53), and subjective sense of smell (pretreatment mean: 2.18, posttreatment mean: 0.65) (47). An evidence-based systematic review of OCS use revealed that there may be significant short-term improvements in subjective and objective measures and a strong recommendation can be made for use in short-term treatment of CRSwNP patients (52).

#### 2.3.3. Exhalation delivery system of fluticasone

One delivery system that improves penetration of corticosteroids is EDS-FLU which consists of a mouthpiece and nosepiece that utilizes the force of exhalation into the mouthpiece to isolate the area for drug deliverance (45). The NAVIGATE I (*n* = 323 patients) and II (*n* = 323 patients) trials were two randomized placebo-controlled, double-blind trials that evaluated the safety and outcomes of EDS-FLU compared to a placebo for CRSwNP patients (53, 54). Baseline endoscopic nasal polyp score was 3.7. At 16 weeks since EDS-FLU was trialed, the mean polyp grade change was −0.96, −1.03, and −1.06 for the 93-µg, 186-µg, and 372-µg groups respectively compared to −0.45 of that in the placebo (53). Pooled data suggest that at 16 weeks follow-up, EDS-FLU treatment is associated with greater improvement in health-related quality of life (assessed by the Short Form Health Survey) compared to placebo (55). Pooled data also demonstrated that for all outcomes assessed at 4 weeks (nasal polyp grade, SNOT-22 scores, patient global impression change, and congestion score), more patients in the EDS-FLU cohort demonstrated pre-defined score improvement than did patients who had a placebo (56).

#### 2.3.4. Corticosteroid-eluting sinus implants

Drug-eluting sinus implants have been shown to improve postoperative outcomes by delivering localized corticosteroids directly into inflamed sinonasal tissue (29). Such implants are available and approved by the FDA for either in-office or intraoperative use. Both mean polyp grade and SNOT-22 scores improved after treatment (57). The RESOLVE I and II trial sought to understand the outcomes and safety of in-office bioabsorbable sinus implants after ESS (28, 58, 59). Pooled data from the two randomized control RESOLVE trials (*n* = 375 patients) found that those receiving mometasone furoate sinus implants and nasal spray had significant improvement in obstructive/congestive symptom score, polyp grade, and ethmoid sinus obstruction than patients who used the nasal spray alone (60). Patients who had the largest improvement in nasal obstruction and congestion were patients without asthma or only one prior ESS (60). The largest treatment effects were seen for patients who underwent ESS <24 months, bilateral nasal polyp grade greater than 5, and patients with moderate to severe allergic rhinitis (60).

#### 2.3.5. Biologics

In 2019, the FDA approved the use of dupilumab, a biologic for CRSwNP treatment (61). Since then, mepolizumab and omalizumab have also been approved for CRSwNP. No head-to-head trials have been performed comparing biologics and additional high-quality evidence is needed. However, a systematic review and meta-analysis of randomized control trials comparing biologics for the treatment of CRSwNP (*n* = 9 studies) suggested that dupilumab was the best biologic option for CRSwNP in terms of safety and efficacy (62). For patients who may have contraindications to dupilumab, omalizumab was second in efficacy in terms of SNOT-22, smell identification, and nasal congestion score whereas mepolizumab was second in efficacy in terms of nasal polyp score (62). Another meta-analysis analyzing similar outcomes found that patients who had dupilumab were associated with a reduced need for surgery, reduced need for OCS, improved smell identification, and improved quality of life scores (63). Trials have demonstrated that dupilumab improved outcomes such as smell, nasal polyp score, and nasal congestion irrespective of surgical history. However, optimal benefits are noted with a shorter duration since the last surgery (64). Omalizumab also showed similar outcomes but had more treatment-related adverse events (63). Randomized-control trials (*n* = 24) demonstrated that omalizumab decreases total nasal endoscopy score at 16 weeks irrespective of allergy status compared to placebo (65). On meta-analysis of the randomized-control trials for omalizumab, the mean change of nasal polyp score was −1.20 (score range of 0–8) with a high certainty of evidence (66). The change in SNOT-22 scores was 15.62 points lower in patients receiving omalizumab, an improvement that was higher than the minimal clinically important difference of 8.9 points (66). Further testing however is necessary to determine the appropriate patient selection and role of IgE levels in treatment outcomes.

Mepolizumab was found to be an efficacious option for patients with OCS-dependent severe eosinophilic asthma with CRSwNP. A randomized control trial (*n* = 16 patients) demonstrated a successful reduction in the OCS dosage of all patients, with 40% even discontinuing OCS by 24 weeks (67). Additionally, significant improvement in nasal polyposis severity compared to placebo was demonstrated with a treatment difference of −1.8 by 25 weeks of treatment (68). Around 50% (*n* = 27 patients) improved by one or more points in total endoscopic nasal polyp score compared to 25% (*n* = 14 patients) of the placebo group (68). SNOT-22 scores also significantly improved for the mepolizumab cohort (51.5 to 28.8) in comparison to the placebo cohort (49.5 to 38.2) by 25 weeks (68). Another randomized control trial found similar results with mepolizumab treatment improving nasal polyp size and nasal obstruction in comparison to placebo and standard of care treatment at 52 weeks (17). The lack of head-to-head studies comparing endpoints of biologics makes it difficult to compare objective results. Nevertheless, mepolizumab has the potential to reduce the burden faced by patients with severe bilateral nasal polyposis.

One important consideration for at-home treatment is compliance. Patients may forget dosages or take medications incorrectly which could minimize the optimal benefits offered. Studies from asthma show that compliance is a problem in 1/3 of patients or more (69, 70). Administration of dupilumab necessitates active patient participation for subcutaneous injections every two weeks. Patients unwilling to or unable to perform self-injections would not be candidates for dupilumab.

#### 2.3.6. ESS

For patients who prefer surgery, ESS may be an option. Interestingly, a recent study regarding patient satisfaction and ESS found that pre-operative expectations were correlated with satisfaction (1). This finding demonstrates that symptom improvement might not necessarily be the most important goal for a patient. It is therefore important to determine what the patient's expectations are and offer appropriate therapies.

For ESS, the recurrence rate ranged from 62–78.9% of CRSwNP patients and the revision surgery rate ranged from 3.5–36.8% depending on the baseline cohort characteristics (71–73). The effect size of biological treatment as most trialed biologics is less than 2 point score when treating patients with nasal polyps scores of 5 to 8. Surgery that is effectively performed reduces the total polyp score to 0 before recurrence, if any, occurs. In terms of symptom improvement, dupilumab in particular is effective in some patients in smell restoration, and this might be superior to what might be achieved by surgery although no randomized clinical trial has compared the two modalities. Recently Miglani et al. compared outcomes of ESS and biologics for CRSwNP. The authors found that ESS and dupilumab had comparable improvement in patient-reported outcome measures and smell identification; however, in terms of polyp size reductions, ESS was superior (74). ESS appeared to offer superior symptom improvements when compared to omalizumab at 24 weeks and mepolizumab at 52 weeks (74). SDM becomes vital in this scenario to decide which option would provide the greatest patient satisfaction and benefit. A patient whose primary goal is the improvement of smell identification might prefer dupilumab or ESS over other options for smell recovery. In general, it is difficult to prognosticate which patient will recover smell, either with surgery or with the use of dupilumab. In contrast, another patient's goal may be the reduction or elimination of nasal polyps for nasal obstruction and in that case, would benefit more from ESS.

Table 4 presents treatment arm outcomes data from recently published phase 3 randomized control trails investigating treatments for CRSwNP. This summarizes comparative efficacy of treatment options.

**Table 4:** CRSwNP treatment efficacy based on randomized control clinical trial data.

	Endpoints	Baseline (mean ± SD)	Time point	Post treatment score (mean ± SD)	LS mean change from baseline (±SE)	Relative change
ESS	NPS	—	Week 24	—	—	—
(*n* = 111)	NCS	2.9 [±0.3]	Week 24	0.9 [±0.9]	−1.9 [±0.09]	−66%
	LK–NP	4.0 [±–]	Week 24	0.9 [±1.2]	−3.1 [±0.14]	−80%
	Loss of smell score	2.7 [±0.7]	Week 24	1.5 [±1.2]	−1.2 [±0.13]	−44%
	SNOT 22 score	56.1 [±19.6]	Week 24	22.9 [±19.6]	−33.3 [±1.8]	−59%
	UPSIT-40 (*n* = 4)	23.3 [±12.5]	Week 24	31.8 [±5.2)	8.5 [±5.2]	36%
	Sniffin’ sticks total (*n* = 34)	13.9 [±6.9]	Week 24	21.1 [±8.3]	7.1 [±1.4]	51%
	Endpoints	Baseline (mean ± SD)	Time point	Post treatment score (mean ± SD)	LS mean change from baseline (±SE)	Relative change
Dupi-24	NPS	5.64 [±1.23]	Week 24	3.75 [±1.98]	−1.89 [±0.14]	−34%
(*n* = 143)	NCS	2.26 [±0.57]	Week 24	0.94 [±0.75]	−1.34 [±0.07]	−59%
	Loss of smell score	2.70 [±0.57]	Week 24	1.35 [±0.99]	−1.41 [±0.07]	−52%
	SNOT 22 score	48.00 [±20.16]	Week 24	18.58 [±14.92]	−30.43 [±1.54]	−63%
	UPSIT-40	14.68 [±8.66]	Week 24	25.39[±9.49]	11.26 [±0.67]	77%
	Endpoints	Baseline (mean ± SD)	Time point	Post treatment score (mean ± SD)	LS mean change from baseline (±SE)	Relative change
Dupi-52	NPS	6.07 [±1.22]	Week 52	3.76 [±2.20]	−2.24 [±0.15]	−37%
(Group A q2w dosing: *n* = 150)	NCS	2.48 [±0.62]	Week 52	1.10 [±0.92]	−1.35 [±0.07]	−54%
	Loss of smell score	2.81 [±0.46]	Week 52	—	—	—
	SNOT 22 score	50.16 [±19.72]	Week 52	21.67 [±19.16]	−29.84 [±1.63]	−59%
	UPSIT-40	13.46 [±8.20]	Week 52	—	—	—
	Endpoints	Baseline (mean ± SD)	Time point	Post treatment score (mean) (Unable to calculate SD)	LS mean change from baseline (±SE)	Relative change
Oma-1	NPS	6.2 [±1.0]	Week 24	5.12	−1.08 [±0.16]	−17%
(*n* = 72)	NCS	2.4 [±0.7]	Week 24	1.51	−0.89 [±0.10]	−37%
	Loss of smell score	2.5 [±0.8]	Week 24	1.94	−0.56 [±0.09]	−22%
	SNOT 22 score	59.8 [±19.7]	Week 24	35.1	−24.70 [±2.01]	−41%
	UPSIT-40	12.8 [±7.9]	Week 24	17.24	4.44 [±0.84]	35%
	Endpoints	Baseline (mean ± SD)	Time point	Post treatment score (Unable to calculate SD)	LS mean change from baseline (±SE)	Relative change
Oma-2	NPS	6.4 [±0.9]	Week 24	5.5	−0.90 [±0.17]	−14%
(*n* = 62)	NCS	2.3 [±0.7]	Week 24	1.6	−0.70 [±0.11]	−30%
	Loss of smell score	2.6 [±0.8]	Week 24	2.02	−0.58 [±0.10]	−22%
	SNOT 22 score	59.2 [±20.5]	Week 24	37.61	−21.59 [±2.25]	−36%
	UPSIT-40	12.8 [±7.6]	Week 24	17.11	4.31 [±0.83]	34%
	Endpoints	Baseline (mean ± SD)	Time point	Post treatment score (Unable to calculate SD)	LS mean from baseline (±SD)	Relative change compared to baseline
Mepo	NPS	5.4 [±1.2]	Week 52	4.5	−0.9 [±1.90]	−17%
(*n* = 206)	NCS	2.67 [±0.24]	Week 42	1.4	−1.26 [±1.03]	−47%
	Loss of smell score	2.88 [±0.24]	Week 52	2	−0.84[±1.08]	−29%
	SNOT 22 score	63.7 [±17.6]	Week 52	34.3	−29.4 [±24.67]	−46%
	Endpoints	Baseline (mean ± SD)	Time point	Post treatment score (Unable to calculate SD)	LS mean change from baseline (±SE)	Relative change
EDS-FLU 373 ug	NPS	3.7 [±0.9]	Week 24	2.3	−1.43 [±0.14]	−39%
(*n* = 80)	NCS	2.29 [±0.43]	Week 4	1.6	−0.70 [±0.11]	−30%
	Loss of smell score	—	Week 4	—	−0.33 [±0.08]	—
	SNOT 22 score	52.4 [±20.07]	Week 24	30.68	−21.72 [±2.07]	−41%
	Endpoints	Baseline (mean ± SD)	Time point	Post treatment score (Unable to calculate SD)	LS mean change from baseline (±SE)	Relative change
MFNS Implant	NPS	5.95 [±0.94]	Week 4	5.39	−0.56 [±1.06]	−9%
(*n* = 80)	NCS	2.4 [±0.5]	Week 4	1.47	−0.93 [±0.80]	−39%
	Loss of smell score	4.1 [±1.4]	Week 4	2.9	−1.2 [±1.66]	−24%

Table adapted from Miglani et al. (74).

—, comparisons were unable to be made due to unavailable data; ESS, Endoscopic sinus surgery; Dupi-24, Dupilumab Liberty NP SINUS-24; Dupi-52, Dupilumab Liberty NP SINUS-52; Oma-1, Omalizumab POLYP-1; Oma-2, Omalizumab POLYP-2; Mepo, Mepolizumab SYNAPSE; LS, Least square; NPS, Nasal polyp score; NCS, Nasal congestion score; LK-NP, Lund-Kennedy Polyp score; SNOT22, Sinonasal Outcome Test 22; UPSIT-40, University of Pennsylvania Smell Identification Test-40; SD, Standard deviation; SE, Standard error; EDS-FLU, exhalation delivery system with Fluticasone; MFNS, mometasone furoate nasal sinus implant; q2w, once every two weeks.

### 2.4. Cost of treatment

CRSwNP accounts for an annual healthcare cost burden of $5.7 billion (75). Additionally, the treatment choices also vary in cost. The financial burden faced by the patient is an important factor in treatment access especially when multiple options are available.

#### 2.4.1. Corticosteroids

Corticosteroid nasal rinses and sprays are often the least expensive option and can be found over the counter. When analyzing the cost of OCS use in CRSwNP, patients receiving OCS had an incremental cost of $4,502 over one year compared to patients who did not receive OCS (75). In low-income families who may not be able to afford a large investment in the short-term, corticosteroids are a more affordable option that can improve everyday quality of life.

EDS-FLU is more expensive than the over-the-counter corticosteroid options without insurance. However, based on insurance coverage, it is possible that this therapy may be more affordable. Atomizers and nebulizers are other affordable options available for patients. When the cost of bronchodilator delivery methods in the Emergency Department setting for asthma was analyzed, the cost for continuous nebulization was $9.66 and for intermittent updraft nebulization was $11.66 (76). Based on healthcare insurance, the total cost for a patient utilizing atomizers and nebulizers may be even lower.

#### 2.4.2. Corticosteroid-eluting sinus implants

A study in the United Kingdom sought to identify the cost-effectiveness of corticosteroid-eluting sinus implants compared to non-corticosteroid-eluting spacers after ESS for postoperative management. The study found that sinus implants were less costly and more effective (77). A similar analysis performed in the United States found that the mean cost for the steroid-eluting and nonsteroid-eluting sinus implant strategies were $1,572.91 and $365.18 respectively (78). Overall, the study determined that mometasone steroid-eluting sinus implant following ESS is a cost-effective measure to prevent postoperative intervention within 60 days after surgery (78). Additionally, other studies have noted that patients with sinus implants had lower healthcare resource utilization over 18 months compared to patients without sinus implants (29). With more effective treatment, fewer resources are utilized and this may be one reason for the results contributing to improved cost-effectiveness.

#### 2.4.3. Biologics and ESS

The cost of ESS and the cost of biologics vary per patient based on insurance claims and status. The annual cost of biologics ranges from $27,800–$31,000. Whereas in 2014, the overall cost of ESS was between $8,200–10,500 (79, 80). When analyzing cost burden and resource utilization, CRSwNP patients undergoing ESS had an incremental cost of $13,532 over one year compared to CRSwNP patients not undergoing ESS (75). A cost-effectiveness analysis for biologic therapies for asthma did not meet effectiveness thresholds and postulated that a 60% minimum reduction in cost would be necessary (81). Currently, sinus surgery is more cost-effective than biologics (82). However, patients with additional medical comorbidities may not be able to elect surgery or may anyway need biologics for the treatment of their comorbid condition. Endpoints for cessation of maintenance treatment, particularly biological therapy have not been defined. Major considerations to continue biologics were proposed as improved quality of life or a greater 50% reduction in systemic corticosteroid use without further surgery (37). Therefore, many patients who find benefits in the use of biologics should continue therapy and continued utilization of biologics likely contributes to the cost burden. Therefore, it is important to understand the patient's budget and other medical conditions when developing individualized medical treatment plans. SDM has been shown to minimize healthcare utilization and has the potential to increase healthcare savings (83, 84).

## 3. Actionable recommendations

• The use of SDM has been shown to offer many clinical benefits to both physicians and patients alike and can serve to improve the quality of care offered to patients with CRSwNP
• Due to the growing number of CRSwNP treatment modalities, variations in treatment cost, and variations in patient goals, physicians should advocate for active patient participation when choosing a treatment.
• No interventional studies have yet been performed to analyze SDM in rhinology (8). Additionally, no studies have been conducted to verify the utility of decisional aids developed for CRSwNP. Addressing these gaps in the literature will help bring SDM to the forefront of strategies that can be utilized for decisions regarding CRSwNP treatment.
• Important factors to discuss with patients diagnosed with CRSwNP as part of an SDM approach include treatment cost, efficacy, risk, delivery, and outcomes.
• For most primary CRSwNP patients that are surgery naïve, current efficacy data and cost-analyses support medical therapy (i.e. topical corticosteroid maintenance therapy with sparing use of oral steroids) followed by ESS. SDM is particularly helpful for patients who have failed medical therapy and complete ESS.

## 4. Discussion

### 4.1. Barriers to SDM

#### 4.1.1. Communication

Communication is an important aspect of the patient-physician relationship. SDM was developed to foster improved communication however presents many barriers that should be acknowledged to offer effective care (85). When used correctly, SDM is advantageous. Likewise, studies have shown that poor techniques lead to poorer patient-reported health outcomes and higher healthcare use (86). Therefore, it is important that proper communication occurs throughout the SDM process.

The disconnect between the physician's explanation and the patient's understanding of treatment is important to identify for many reasons. One, patients might have a different expectation of the outcome of a procedure which, if not identified, may lead to dissatisfaction with treatment (1). Second, patient health literacy is important and an incorrect understanding of how the treatment should be used might lead to decreased compliance and subsequently lower treatment response. Patient understanding of their disease and disease process empowers patients to advocate for themselves, actively participate in care, and improves health outcomes (87).

The patient's preferred decision-making control exists in a spectrum. Some patients prefer to make decisions themselves after reviewing options, regardless of a physician's recommendation. Others prefer that medical decisions should be made by their physician (9). These styles can be summarized into four main categories- paternalism, deferential, participatory, and directed (88). Studies have found that patients who are actively involved in decision-making derive the most clinical benefit, even in patients who prefer to take a passive approach (89). A focus group study examining the reasons for passive engagement found that fear of being categorized as a “difficult” patient and the authoritarian style of a physician's presence are contributing factors (90).

Socioeconomic barriers might also exacerbate such fears and studies have found that vulnerable patients, such as those who are immigrants, have less education, or are elderly, reported lower interest in SDM (88). Alleviating some of the barriers faced by patients when communicating with physicians and empowering collaboration may help patients proactively engage in SDM and subsequently improve outcomes.

#### 4.1.2. Implementation

One of the most cited barriers in SDM implementation is time. Implementation of SDM with decision aids was found to increase each visit by 2.6 min which when compounded with the number of patients during a clinic day can result in a significantly increased workload (91). However, no conclusive evidence of increased time requirement was seen in the utilization of SDM as a whole (9). In addition, the long-term impacts of SDM are thought to reduce the total amount of healthcare utilization. This is an association that might possibly save time in the long run with reduced clinic visits (83). The large variety of treatment options for CRSwNP allows the opportunity to utilize SDM in determining a patient-specific treatment plan agreeable to all parties. Addressing the barriers to successful employment of SDM is vital for the implementation of SDM and optimal outcomes.

In order to implement SDM for CRSwNP globally, several other considerations specific to the patient population and country must be incorporated. Although several biologics and sinus implants have been approved for use in the United States, different regulatory agencies oversee the verification of these treatment modalities worldwide. For example, absorbable sinus stents are not available in many European countries. Healthcare insurance status, coverage, and reimbursement policy varies between each country and may also impact the treatment options available or are affordable. Socioeconomic challenges unique to each country may also affect the availability of treatment options for patients. In low-income countries, several interests such as vaccination programs, communicable disease treatment programs, and nutritional aids, must compete for the limited healthcare resources available. Disparities in a country's education opportunities and cultural and religious predispositions may also impact treatment adherence. These considerations may vary extensively by country, ethnicity, and race. SDM provides a framework that healthcare workers should appropriately adapt to each circumstance for effective patient care.

### 4.2. Role of decision aids

Decision aids act as informative tools that can aid in SDM. Studies in other fields have demonstrated that decision aids serve to improve patients' healthcare literacy, reduce decisional conflict, and stimulate active patient participation (92, 93). However, the role of decision aids in rhinology is limited. Additionally, there is great variability in formats developed ranging from in-consultation paper aids to electronic aids reviewed outside of a consultation. A systematic review that aimed to determine the effectiveness of decision aids in a primary care setting (*n* = 24 studies) found multiple types of decision aid formats; However, there were no differences when comparing strategies (93).

In terms of CRSwNP, the ARS has developed a decision aid with information about FDA-approved treatment options available to download online. The goal of the online pamphlet is for patients to read through, respond to statements based on preferences, and then have a joint discussion with a physician about treatment options. The brochure explains the initial and recurrent treatment options for CRSwNP with charts explaining treatment use, risks, advantages, and cost (94). In early 2022, The American College of Allergy, Asthma, and Immunology in partnership with ARS, developed additional decision aids for CRSwNP. This includes a CRSwNP toolkit for allergists to help answer commonly asked questions, an interactive electronic SDM tool for patients, and an instructional video for allergists containing tips on implementing SDM into their practice for CRSwNP (95).

Although there has been much activity in developing and advocating decision aids in CRSwNP, no studies have been performed demonstrating the efficacy and outcome of these specific decision aids for patients. Future studies on decision aids in CRSwNP are needed to demonstrate evidence-based data on the validity of these aids. This would be the next step to developing widespread utilization of decision aids in common rhinology practice.
